# Determining the In Vivo Efficacy of Plant-Based and Probiotic-Based Antibiotic Alternatives against Mixed Infection with *Salmonella enterica* and *Escherichia coli* in Domestic Chickens

**DOI:** 10.3390/vetsci10120706

**Published:** 2023-12-15

**Authors:** Ádám Kerek, Ábel Szabó, Péter Ferenc Dobra, Krisztina Bárdos, László Ózsvári, Péter Fehérvári, Zsófia Bata, Viviána Molnár-Nagy, Ákos Jerzsele

**Affiliations:** 1 Department of Pharmacology and Toxicology, University of Veterinary Medicine, István Street 2, 1078 Budapest, Hungary; abelszabo02@gmail.com (Á.S.); jerzsele.akos@univet.hu (Á.J.); 2 National Laboratory of Infectious Animal Diseases, Antimicrobial Resistance, Veterinary Public Health and Food Chain Safety, University of Veterinary Medicine, 1078 Budapest, Hungary; bardos.krisztina@univet.hu (K.B.); ozsvari.laszlo@univet.hu (L.Ó.); 3 Department of Pathology, University of Veterinary Medicine, 1078 Budapest, Hungary; dobra.peter@univet.hu; 4 Department of Veterinary Forensics and Economics, Institute of Economics and Biostatistics, University of Veterinary Medicine, 1078 Budapest, Hungary; 5 Department of Biostatistics, Institute of Economics and Biostatistics, University of Veterinary Medicine, 1078 Budapest, Hungary; fehervari.peter@univet.hu; 6 Dr. Bata Zrt., 2364 Ócsa, Hungary; sbata@drbata.com (Z.B.); research@drbata.com (V.M.-N.)

**Keywords:** *Salmonella enteritidis*, antibiotic alternatives, laying hens, *Trigonella foenum graecum*, *Triticum aestivum*, *Curcuma longa* L., probiotics

## Abstract

**Simple Summary:**

Antimicrobial resistance is recognised as one of the most important animal and public health challenges of our time, and we all have a responsibility to successfully tackle it. With the number of effective antibacterial compounds decreasing at an alarming rate, alternative methods to fight bacteria that cause infections are needed. Many natural compounds produced by plants have been shown to have in vitro antimicrobial properties. In our study, we tested feed supplements containing various plant-derived active substances that can reduce the emergence and spread of *Salmonella* infection, thereby reducing both economic and health risks. During the experiment, we monitored the tested animals’ *Salmonella* shedding, the manifestation of clinical signs following infection, the consumption of feed, body weight gain, and pathological and pathovegetative changes. A statistical analysis of the obtained data revealed that fenugreek extract is the most promising feed supplement in terms of both the natural indicators and the pathobiological parameters that determine them. It would be worth considering combining fenugreek extract with turmeric extract, conducting further in vivo studies to explore possible interactions between the two, and performing a dose-response study to determine the optimal dosage.

**Abstract:**

Restrictions on the use of antimicrobial compounds have led to a surge of interest in alternative solutions, such as natural, plant-based compounds. In our study, we investigated the efficacy of three feed supplements containing different additives, namely, probiotics (*Lactobacillus* spp., “Test substance A”), turmeric (*Curcuma longa* L., “Test substance B”), and fenugreek (*Trigonella foenum graecum*, “Test substance C”). In the experiment, we tested 180 birds of the Bábolna Tetra-SL laying hybrid breed that were infected with *Salmonella enteritidis* strains. The birds were randomly divided into six groups: three groups treated with the different additives, a negative control group, a positive control group, and an antibiotic-treated group using enrofloxacin. We examined the maturation and the time course of shedding of *Salmonella*; at the end of rearing, pathological and histopathological examinations were performed. When *Salmonella* was isolated from the cloacal swab samples, the enrofloxacin-treated group had a high number of animals shedding *Salmonella* by day 9, which was like the group treated with test material C. The greatest reduction in *Salmonella* shedding was observed in the groups treated with test materials A and B. In terms of pathological parameters, villus length and crypt depth were significantly better in the group treated with test material C compared to the positive and negative controls, and when comparing the body weight of the tested animals, the group treated with test material B had a significantly larger absorption surface area compared to the positive control group. Overall, the supplement with test material C proved to be the most effective. In the future, it is worthwhile to investigate the combination of the tested active substances for their possible synergistic effects and to perform a dose-response study to select the optimal dosage.

## 1. Introduction

Excessive and unregulated use of antibiotics in recent decades and societal and economic trends have significantly accelerated the selection and spread of resistant bacteria, resulting in a significant increase in the number of related deaths [1]. Currently, around 700,000 deaths per year are linked to antimicrobial resistance (AMR), which, according to the most conservative estimates, could reach 10 million per year by 2050 if we continue to use antibiotics at similar rates and fail to keep pace with the development of new therapies and active substances [2].

In Europe, penicillin and tetracycline are the most widely used active substances for veterinary purposes. In some countries, the use of these two groups can be up to 30 times higher than that of other groups of active substances [3]. In terms of average antibiotic use per livestock sector, the pig sector is the leader (172 mg/p.u.), followed by the poultry sector (148 mg/p.u.) and the cattle sector (45 mg/p.u.) [4].

The genus *Salmonella*, belonging to the family Enterobacteriaceae, is composed of rod-shaped, Gram-negative, facultative anaerobic bacteria [5]. The *Salmonella* serotypes that cause human disease are classified into a group that causes human-only bacterioses (*S. Typhi* and *S. Paratyphi*) and a group of thousands of different serotypes that cause non-typhoidal diseases [6]. Human salmonellosis is a complex, potentially fatal zoonotic disease that includes all *Salmonella* serotypes. Poultry farming is a major source of these infections, accounting for almost 50% of cases of salmonellosis in the European Union in the 2000s and in Asian countries in the 2010s [7].

*Escherichia coli* prefers aquatic environments and belongs both to the animal and to the human microbiome [8]. Although most strains of *Escherichia coli* are very common and commensal, some strains can cause serious infections ranging from gastroenteritis to extraintestinal inflammation [9]. Colibacillosis can be found worldwide, causing significant economic damage and increasing the amount of antibiotics used in poultry farming [10]. Avian pathogenic *Escherichia coli* (APEC), which can cause serious losses in the poultry industry, can affect many organ systems, e.g., the respiratory, digestive, reproductive, or locomotor systems, or may occur locally, as in the case of yolk sac infections and omphalitis, and in many cases secondary infections also occur, further aggravating the disease [11].

Several types of food have been associated with cases and outbreaks of salmonellosis, but in the European Union, 45.6% of reported cases in 2018 were associated with eggs or food containing eggs [12]. The prevalence of *S. enteritidis* in laying hens producing eggs for consumption is directly related to the prevalence of human infection, and the pathogenic strains are often genetically identical [13].

Resistance to chloramphenicol was described among *Salmonella* strains in Mexico as early as 1972, causing more than 10,000 cases [14]. Subsequently, in the 1980s, strains also emerged that became resistant to three commonly used antibiotics (ampicillin, chloramphenicol, and sulfamethoxazole/trimethoprim) in the treatment of salmonellosis [15]. Newer strains resistant to ciprofloxacin, nalidixic acid, azithromycin, and cephalosporins have also emerged [16]. The management of infections caused by multiresistant strains is becoming an increasing challenge for humankind, and this has prompted researchers to search for alternative solutions [17].

For centuries, humans have been using various products of plant origin for healing. It is estimated that there are around half a million plant species on the planet, of which only 1% have been studied for their bioactive compounds (phytochemicals) [18]. Plants are capable of synthesising aromatic compounds, most of which are phenols or their oxygen-substituted derivatives. To date, more than 12,000 different compounds have been isolated from plants, but it is estimated that this is less than 10% of the total amount of compounds found in plants [19], which represents a huge potential for the discovery of new bioactive agents [20]. In particular, flavonoids have been shown [21] to be effective against a wide spectrum of microorganisms [22,23], mainly due to their complexing and membrane-damaging properties [24,25].

In recent years, the use of herbal products to enhance yield in poultry production has increased dramatically. Among such herb products, thyme, oregano, rosemary, marjoram, yarrow, garlic, ginger, green tea, black cumin, coriander, cinnamon, and their mixtures have been shown to be useful for yield enhancement [26], while beneficial effects have also been reported for probiotics [27].

Probiotics can protect the intestinal epithelium from pathogenic bacterial colonisation by reducing the adhesion and invasion capabilities of pathogens, making them suitable for preventing enteric diseases such as salmonellosis [28]. There are several different mechanisms involved in preventing pathogenic colonisation, such as competing for the same receptor sites, limiting nutrients, or producing antimicrobial metabolites [29]. Several different bacterial strains have been shown to be effective in reducing the incidence of *Salmonella* infection and improving the gut health of chickens [30,31,32].

Natural bioactive substances are capable of modulating gut microbiota in a symbiotic equilibrium, thereby enabling the intestinal tract to withstand both infectious and non-infectious stressors [33], improving productive performance, bone mineralisation, and intestinal integrity [34]; and helping to reduce the incidence of *Salmonella* spp. from commercial turkey operations [35,36]. Therefore, the aim of the present study is to determine the efficiency of different plant-based feed additives in reducing the risk of *Salmonella* infection, thereby reducing its economic damage and its role as a human health risk.

## 2. Materials and Methods

### 2.1. Animals, Diets, and Experimental Design

The animal experiments were conducted in strict accordance with the guidelines of Hungarian Government Decree No. 40/2013 (II. 14.) and were approved by the ethical committee of the Veterinary University of Budapest (Licence No. PE/EA/01448-6/2022), and the severity of the animal experiment was categorised as mild.

A total of 180 Bábolna Tetra SL laying hybrid day-old chicks were involved in the experiment, which received NOBILIS^®^ RISMAVAC + CA126 (Marek’s disease), Nobilis ND C2 (fowl influenza), and Poulvac IB Primer vaccine (infectious bronchitis) in the hatchery. Upon arrival, animals were randomly allocated into 18 groups with a sex ratio of 1:1. A total of 10 animals were placed in each cage. Experiments were conducted in the animal house of the Veterinary University of Budapest. During the experiment, all temperature and lighting regimens were kept according to the husbandry technology and to the breeds specifications. The animals were housed in conventional cages (90 cm × 112 cm × 55 cm). Each repetition was housed in a separate cage to avoid direct physical contact and minimise cross-contamination. Each pen was equipped with a stainless-steel feeder and a nipple drinker with *ad libitum* access to feed and water through the experiment. The animals received fresh drinking water daily. The animals were fed pre-starter lay hybrid feed in weeks 1–3 and starter egg hybrid feed in weeks 4–6. The composition of the feed as well as the nutritional parameters of the feed were detailed in Supplementary Tables S1 and S2.

The effects of six treatments were compared in the experiment, with each diet fed to three replicates using a double-blind test. Experimental groups received different diets: negative control—basal diet; positive control—basal diet with challenge; Enrofloxacin—basal diet with antibiotic treatment in water (Baytril 100 mg/mL, 10 mg/kg bw as described in the instructions of use); Group A—basal diet supplemented with 1 kg/T prototype feed additive containing probiotics (*Lactobacillus* and *Bifidobacterium*); corncob; and wheat bran; Group B—basal diet supplemented with 1 kg/T prototype feed additive containing turmeric extract; wheat germ; and chicory root; and Group C—basal diet supplemented with 1 kg/T prototype feed additive containing fenugreek extract; copper chelate; and chicory root (Supplementary Table S3). All groups, except for the negative control group, received a mixed infection with *Salmonella enteritidis* and *Escherichia coli* by oral gavage on day 3 of age.

### 2.2. Mode of Infection and Monitoring of Animals

Mixed infection using *Salmonella enteritidis* and *Escherichia coli* strains isolated from clinical cases, grown on tryptone-soy agar (TSA; Biolab Zrt., Budapest, Hungary). For this purpose, 18–20 colonies (grown at 37 °C on TSA plates for 18 h) from *Salmonella enteritidis* and *Escherichia coli* were inoculated into 150–150 mL of tryptone soy broth separately (TSB; Biolab Zrt., Budapest, Hungary) on the day of infection and were incubated at 37 °C for 4 h to reach the desired colony forming unit (CFU) (10^8^/mL), similarly to the work by Wu et al. 2020 [37]. Preliminary in vitro studies determined that a 4 h incubation time was sufficient to reach 10^8^ CFU/mL in TSB at 37 °C. In the mixed infection, these microorganisms were able to infect animals and also produce some minor to moderate clinical signs (anorexia, diarrhoea, and apathy). For the determination of the exact microbial count, a ten-fold dilution series was prepared. From each dilution, 50 µL was pipetted to TSA plates in triplicate and incubated at 37 °C for 24 h, and the CFU of the fermentation broth was determined using a colony counting technique by averaging the three parallels. Mixed infection was performed on the animals numbered 1 to 150 (every animal except the negative control group) using a biopsy probe with 1 mL of *Salmonella enteritidis* (6 × 10^8^ CFU) and an additional 1 mL of *Escherichia coli* (2.2 × 10^8^ CFU). A mixed infection with *Escherichia coli* was necessary to provide the optimal circumstances for these pathogens, specifically to ensure the successful colonisation of *Salmonella* bacteria. However, when evaluating the results, it should be taken into account that the *Escherichia coli* infection was also involved in the development of clinical symptoms.

The tested animals were monitored daily for clinical signs of infection (change in faecal consistency, bloody faeces, cloacal area, uric acid build-up, limping, flapping, cowering, and leg ends), and if there was mortality, we measured the weight of the dead animals and carried out further investigations by means of necropsy.

### 2.3. Data Collection

Cloacal swab samples were collected at different time points from each animal, first on the day of infection and then on days 3, 5, 9, 16, 23, 30, and 37 post infection. The swab samples were suspended in Rappaport Vassiliadis (Biolab Zrt., Budapest, Hungary) broth (3 mL/tube) and incubated for 24 h in a 41 °C thermostat. Subsequently, 50 µL/sample were spiked onto Rambach agar (Chebio Ltd., Budapest, Hungary) and incubated for another 24 h at 41 °C, with the temperature controlled using a thermostat. Finally, the growth of *Salmonella* was judged as positive or negative. In cases where the *Salmonella* identification was ambiguous (i.e., the colour of the colony on the Rambach agar was not clearly red), samples were further inoculated on XLD agar (Biolab Zrt., Budapest, Hungary) and *Salmonella*-selective agar (Biolab Zrt., Budapest, Hungary). The shedding of *Escherichia coli* was not measured.

Body weight was measured weekly for 6 weeks for each individual animal. Daily feed consumption was measured per group via back-measuring. At the different measurement times, the difference between the feed given and the feed left over was measured, giving us the exact amount of feed consumed, and then we divided by the number of chickens in the cage for each group. Finally, the feed conversion ratio of the different groups was calculated by dividing the daily feed consumption by the daily weight gain to obtain a proper picture of the possible effects of the additives on the digestive tract and feed utilization.

### 2.4. Pathology and Pathological Histology

At the end of the experiment, when the tested animals were 42 days old, they were euthanised (Euthasol 40% injection A.U.V., 100–200 mg/kg body weight dose intravenously). Pathological examination was performed on each animal according to the rules for diagnostic dissection of birds (external examination and internal examination per organ system). Histopathological examinations were performed on two animals per group using the ileal intestinal tract, with three parallel measurements performed for each sample. Crypt depth, villus thickness, and villus diameter were measured, and the ratio of crypt depth to villus length was calculated. Finally, the size of the absorption surface was determined. To calculate the absorption area, the formula (2π) × (VW/2) × (VH) = π × VW × VH was used, where π = 3.14, VW = villus diameter, and VH = villus length [38].

### 2.5. Statistical Method

The effects of each treatment on weight gain and feed consumption were examined at each time point and compared to the positive and negative control groups, taking into account the effects of diet. We examined the villus length, crypt depth, villus diameter, ratio of villus length to crypt depth, and the size of the absorptive surface of the ileum of each group. The results were statistically analysed using the R programme version 4.3.0 [39], using one-way ANOVA [40] to determine the ratio of feed consumption to feed conversion and a linear model [41] for the other parameters.

## 3. Results

### 3.1. Preparation of the Challenge Bacteria

After 4 h of incubation of the *Salmonella enteritidis* and *Escherichia coli* strains that would be used for infection, the CFU count was calculated by preparing a 10-fold dilution series. It was found that the microbial count inoculated with the bacteriological probe was 6 × 10^8^ CFU for *Salmonella enteritidis* and 2.2 × 10^8^ CFU for *Escherichia coli*.

### 3.2. Mortality

During the experiment, a total of 16 animals out of the 180 animals died following the infection challenge. The distribution of mortality was as follows: two animals from the group treated with test material A, six animals from the group treated with test material B, five animals from the group treated with test material C, one animal from the antibiotic-treated group, one animal from the positive control group, and one animal from the negative control group. The chin drops observed in some groups were indicative of a residual rockworm incubation weakness that is often diagnosed in chickens. In the negative control group, no pathological abnormalities other than scours were observed. In the infection-challenged groups, similar levels of lesions (typhlitis, pneumonia, splenomegaly, rhinitis, fibrinous pericarditis, and tubular renal lesions) were observed, indicating successful infection and colonisation by *Salmonella* and *Escherichia coli*.

### 3.3. Salmonella Shedding

**Figure 1:**
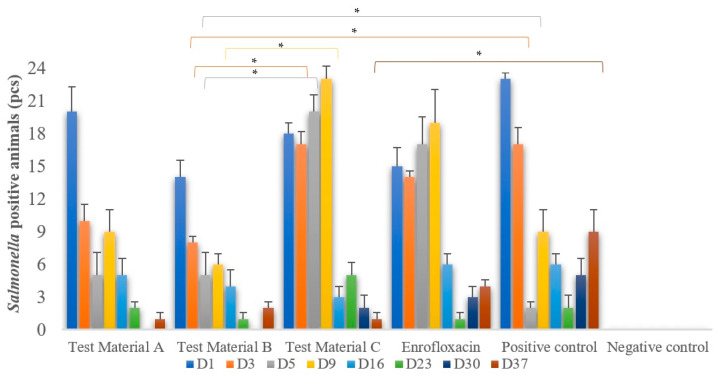
Cloacal swab samples were taken at different time points after infection. *Salmonella enteritidis* enrichment was performed and then isolated on selective agar. The figure shows the number of chickens that excreted positive *Salmonella* and is grouped by treatment. Individual columns are linked, with asterisks above them indicating significant differences. Enrofloxacin treatment alone is not able to significantly reduce or eradicate *Salmonella* shedding. Test material B (*p* = 0.0404) on day 3 and test material C (*p* = 0.0419) on day 37 are the most promising, significantly reducing shedding when compared to the positive control group. * *p* = 0.01.

The results of *Salmonella* isolation from the cloacal swab samples of each treatment group are shown in Figure 1. The negative control group remained negative throughout this study. The positive control group had the highest levels of *Salmonella* shedding. In comparison, the enrofloxacin antibiotic-treated group had high levels of *Salmonella* shedding up to day 9 post-infection, and this was similar for the group treated with test material C. Treatments with test material A and test material B extracts were the most effective in reducing the rate of *Salmonella* shedding. Significant differences in the number of animals shedding *Salmonella* were observed on day 3 between the groups treated with test material C and test material B (*p* = 0.0404) and between the positive control group and the group treated with test material B (*p* = 0.0404); on day 5, significant differences were observed between these same groups, with equal *p*-values. On day 9, significant differences were observed between the groups treated with test material C and test material B (*p* = 0.0225), and on day 37, between the positive control group and the group treated with test material C (*p* = 0.0419).

### 3.4. Body Weight

**Figure 2:**
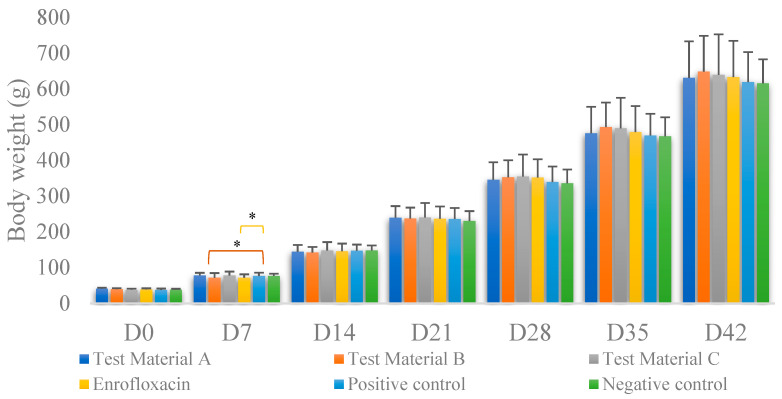
Body weight gain per treatment on each measurement day. Individual columns are linked, with asterisks above them indicating significant differences. For body weight, we found significant differences between the positive control group and the group treated with test material B (*p* = 0.0224) and between the positive control group and the group treated with enrofloxacin (*p* = 0.0276) only immediately after infection. * *p* = 0.01.

The average of individual weights in each group for each day of measurement is shown in Figure 2. The groups showed no significant difference in weight gain compared to either the positive or negative control groups (*p* > 0.05). Significant differences were observed only on day 7 of life (Supplementary Figure S1 and Table S4). Compared to the positive control group, there was significantly less weight gain in the groups treated with test material B (*p* = 0.0224) and enrofloxacin (*p* = 0.0223); when compared to weight gain in the negative control group, there was an increase in weight gain in the groups treated with test material B (*p* = 0.0275) and enrofloxacin (*p* = 0.0276).

### 3.5. Trends in Feed Consumption and Feed Conversion Ratio

The feed consumption on day 10 was higher in the group treated with enrofloxacin than in the group treated with test material B (*p* = 0.0422) and higher in the group treated with test material B than in the negative control group (*p* = 0.0335); on day 13, it was higher in the group treated with test material B than in the positive control group (*p* = 0.0360) and the negative control group (*p* = 0.0335); on day 17, it was higher in the group treated with test material B than in the positive control group (*p* = 0.0360). On day 36, significant differences were found between the group treated with enrofloxacin and the group treated with test material B (*p* = 0.0349), between the groups treated with test materials C and B (*p* = 0.0019), between the group treated with test material B and the negative control group (*p* = 0.044), and between the group treated with test material B and the positive control group (*p* = 0.0023). On day 38, the groups treated with test materials C and B also differed significantly (*p* = 0.0083) based on one-way analysis of variance.

The individual feed consumption on day 22 differed significantly between the group treated with test material C and the negative control group (*p* = 0.0289). On day 27, there were significant differences between the groups treated with test materials C and B (*p* = 0.0353) and between the group treated with test material B and the negative control group (*p* = 0.0095). On day 29, the groups treated with test materials C and B differed significantly (*p* = 0.0353). On day 29, one-way analysis of variance showed a significant difference between the group treated with test material B and the negative control group (*p* = 0.0347) and between the groups treated with test materials A and C (*p* = 0.0116) on day 36.

**Figure 3:**
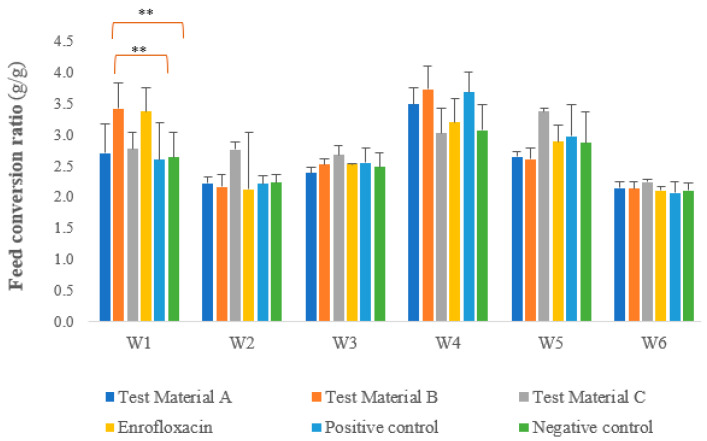
Feed conversion ratio in each week per group per day, with significant differences between the groups treated with the test materials and the positive and negative control groups marked. Other statistically significant differences are listed in the text and are omitted from the figure for simplicity. For feed conversion, we observed a significant negative difference between the positive control group and the group treated with test material B (*p* = 0.0013) and between the negative control group and the group treated with test material B (*p* = 0.0017) during the first week after infection. ** *p* = 0.001.

We calculated the species ratio as the ratio of the individual feed consumption rate to the weight gain rate. Figure 3 shows the feed utilisation measured each week, taking into account daily feed consumption and weight gain. In terms of specific weight, the results for week 1 were as follows: significant differences were observed between the groups treated with test materials C and B (*p* = 0.0077), between the group treated with test material B and the negative control group (*p* = 0.0017), between the group treated with the test material B and the positive control group (*p* = 0.0013), between the groups treated with test materials A and B (*p* = 0.0040), between the groups treated with test material C and enrofloxacin (*p* = 0.0134), between the group treated with enrofloxacin and the negative control group (*p* = 0.0030), between the group treated with enrofloxacin and the positive control group (*p* = 0.0022), and between the groups treated with test material A and enrofloxacin (*p* = 0.0072). In week 5, one-way analysis of variance with a one-tailed test (*p* = 0.0305) showed significant differences between the groups treated with test materials C and B (*p* = 0.0305) and between the groups treated with test materials A and C (*p* = 0.0455).

### 3.6. Pathological Histology

**Figure 4:**
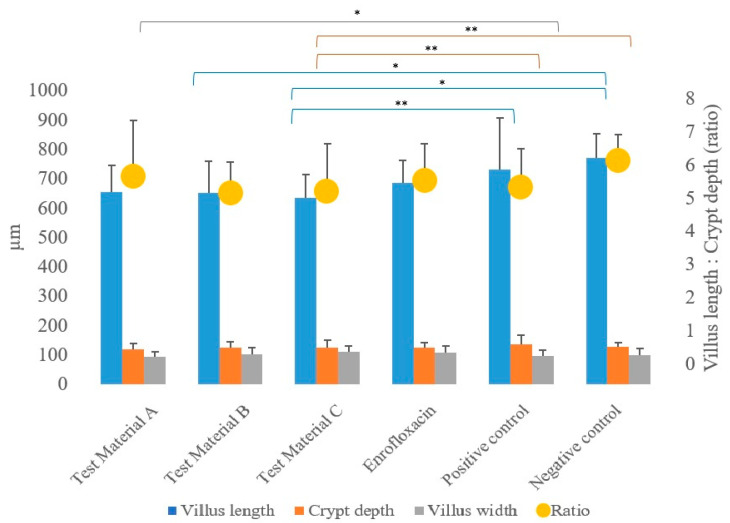
Average of each treatment group as a function of the measured pathogenetic parameters: villus length, crypt depth, villus width, and the calculated ratio of villus length to crypt depth. When the linear modelling statistical method was used to examine the pathological parameters in comparison to the positive control group, significant differences were observed in the group treated with test material C (*p* = 0.0037) for villus length and in the group treated with test material C (*p* = 0.0046) for crypt depth. ** *p* = 0.001; * *p* = 0.01.

The villus length, crypt depth, villus diameter, and ratios of villus length to crypt depth measured at the jejunum intestinal section during the pathological examinations are presented in Figure 4; the results are presented by treatment group.

In the statistical analysis (using a linear model) of pathogenetic parameters, significantly longer villus length (Supplementary Table S5) was measured in the group treated with test material C (*p* = 0.0037) compared to the positive control group (*p* = 0.0037), and the same was observed in the negative control group (*p* = 0.0202). When comparing the groups that received treatments to the negative control group, a significantly shorter villus length was observed for the group treated with test material B (*p* = 0.0372).

In terms of crypt depth (Supplementary Table S6), compared to the positive control, the group treated with test material C (*p* = 0.0046) had significantly shallower crypts. When comparing the groups that received treatments to the negative control group, a significantly higher value for crypt depth was also observed for the group treated with test material C (*p* = 0.0039).

However, the ratios of villus length to crypt depth showed no significant difference (*p* > 0.05) compared to the positive control group for any of the treated groups for both sexes, but were significantly lower (*p* = 0.0310) for the group treated with test material B compared to the negative control group (Supplementary Table S7).

In terms of villus width, the villus width was significantly (*p* = 0.0276) narrower in the test material A-treated group compared to the positive control group. There were no differences between the groups that received treatments compared to the negative control group.

No significant differences were observed in terms of the absorption surface area calculated from the measured parameters for any of the treated groups when compared to the positive control group and the negative control group.

**Figure 5:**
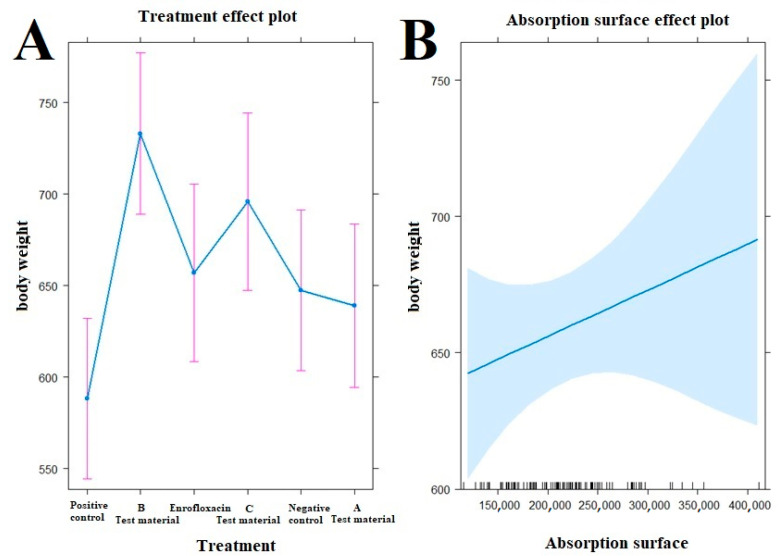
Examination of the relationship between treatments ((**A**), left side) and absorption surface area ((**B**), right side) on the evolution of animal body weight by treatment. The test material B-treated group (*p* < 0.0001), the enrofloxacin-treated group (*p* = 0.0400), and the test material C-treated group (*p* = 0.0015) have significantly better final body weight compared to the positive control group (**A**). Compared to the negative control group, a significantly better final weight was observed in the group treated with test material B (*p* = 0.0078).

When the correlations between body weight and treatment-related absorption surface area were examined (Figure 5), the test material B-treated group (*p* < 0.0001), the enrofloxacin-treated group (*p* = 0.0400), and the test material C-treated group (*p* = 0.0015) had significantly better final body weight compared to the positive control group. Compared to the negative control group, a significantly better final weight was observed in the group treated with test material B (*p* = 0.0078).

**Figure 6:**
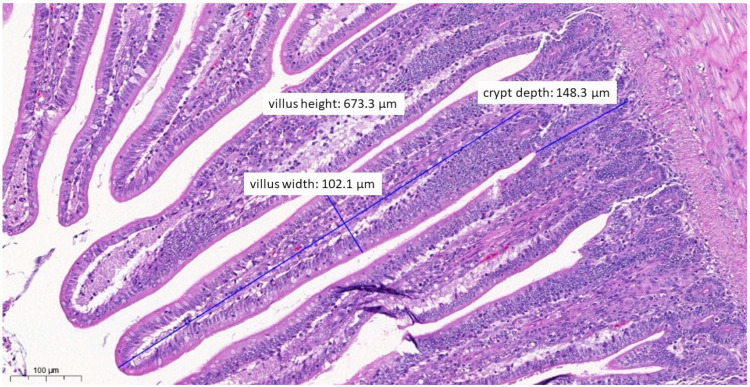
Ileal morphology measurements of chicken number 11 from the group treated with test material A (H&E, 130×). In this case, among the measured parameters, only the villus width (102.1 µm) showed a significantly wider villus compared to the negative control group (*p* = 0.0276).

**Figure 7:**
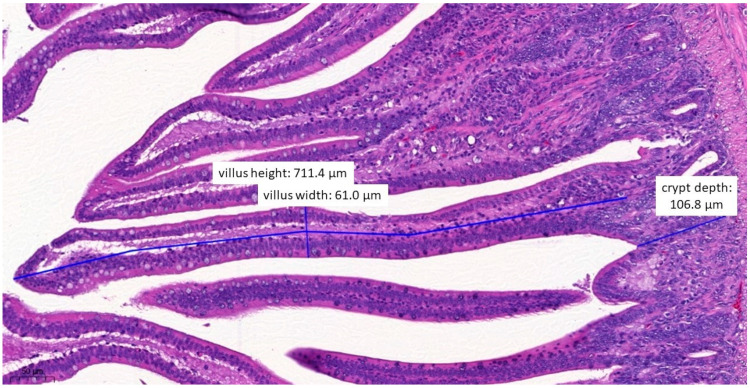
Ileal morphology measurements of chicken number 37 from the group treated with test material B (H&E, 160×). In this case, the villus length (height) is significantly shorter (711.4 µm) compared to the negative control group (*p* = 0.0372).

**Figure 8:**
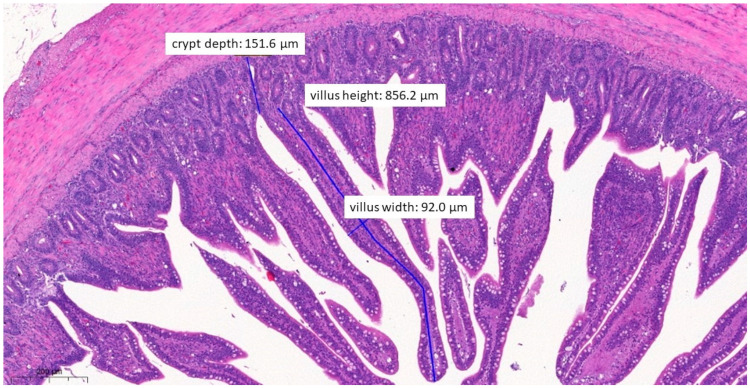
Ileal morphology measurements of chicken number 138 from the group treated with test material C (H&E, 70×). In this case, the villus length (height) is significantly longer (856.2 µm) compared to the positive control group (*p* = 0.0037). The crypt depth is also less deep (151.6 µm) compared to the positive control group (*p* = 0.0046).

**Figure 9:**
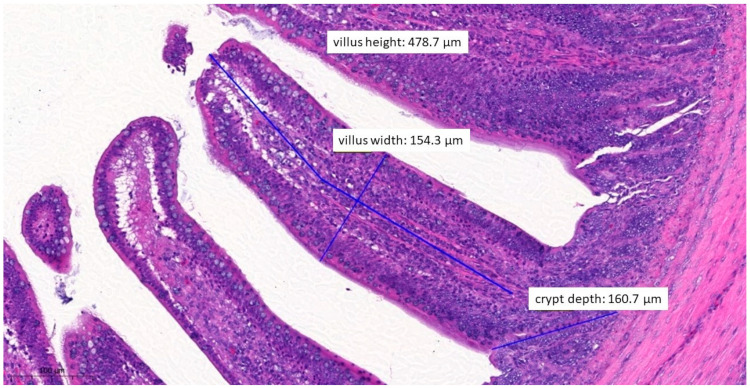
Ileal morphology measurements of chicken number 75 from the positive control group (H&E, 150×). The longer villus and deeper crypts indicate significant regeneration processes following an infection, compared to the groups that received treatments.

**Figure 10:**
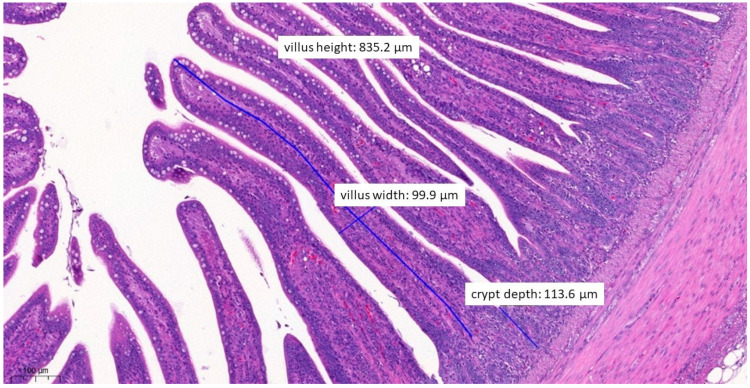
Ileal morphology measurements of chicken number 179 from the negative control group (H&E, 90×). For the negative control group, both the length of the villus and the depth of the crypt are ideal, with a ratio of 1:7, which is the most common healthy ratio described in the literature.

In terms of histopathological images, the group fed with test material A (Figure 6) had, on average, the longest villus, the shallowest crypt depth, the narrowest villus, and the best ratio of villus length to crypt depth among the three treatment groups. The group fed with test material B (Figure 7) had, on average, the deepest crypts. The group fed with test material C (Figure 8) had, on average, the shortest villus but also the widest villus and thus the largest absorption surface area. When averaged across the positive control group (Figure 9), the longer villus and deeper crypts indicate significant regeneration processes following an infection. Regarding the mean of the negative control group (Figure 10), we observed the most favourable villus-length-to-crypt-depth ratio.

## 4. Discussion

In this study, a challenge model was established with *Salmonella enterica* and *Escherichia coli* strains. Manifestations of clinical signs were mainly seen in terms of changes in faecal consistency, with bloody faeces observed in several cases. In most cases, the cause of death following an infection was due to incubation weakness resulting from residual scours. In all groups except the negative control group, pathological lesions at death were presumably attributable to infection. A statistical limitation of our study is the epidemiological independence of each bird group.

The number of positive *Salmonella* samples re-isolated from the tested animals decreased progressively over time. Of the treated groups, the groups treated with probiotics and turmeric extract showed the greatest reduction in the number of animals shedding *Salmonella*. In the enrofloxacin-treated group, the number of animals shedding *Salmonella* was high up to the 9th day post-infection, probably due to the fact that most of the bacteria became resistant to enrofloxacin, although it was found susceptible on preliminary examination. As a consequence, this antibiotic was only effective against a certain percentage of bacteria, whereas in the turmeric-treated group, a beneficial reduction was seen from the beginning as the turmeric additive was able to kill bacteria resistant to enrofloxacin. Varmuzova et al. fed broiler chickens with a feed supplement containing turmeric and found that detectable *Salmonella* levels were significantly reduced only by day 14 after treatment when compared to the controls [42], with a significant reduction in shedding after a similar time interval. Probiotics have been shown to reduce *Salmonella* colonisation in chickens in several cases, including in a study conducted by Pascu et al. on *Lactobacillus salivarius* [43]. Also, Luoma et al. found that feeding *Lactobacillus reuteri* significantly reduced the duration of *Salmonella* shedding [44], and Fernandez et al. reported that mannose oligosaccharide fed as a prebiotic contributed to increased colonisation of *Bifidobacterium* and *Lactobacillus* strains in the caecum, thereby significantly reducing *Salmonella* [45]. Tabashsum et al. showed a significant reduction in *Salmonella* shedding in *Lactobacillus casei*-treated groups compared to controls [46]. Several other plant extracts have been shown to have effective *Salmonella*-reducing effects. Robinson et al. described a reduction in *Salmonella* of up to two log CFU (99%) when *Salmonella*-infected chicken meat was dipped in garlic and ginger oil [47]. Nair et al. demonstrated a pronounced bactericidal effect against *Salmonella* spp. using extracts of Anatolian bocort and Indian cumin at a concentration of 12.5 mg/mL [48]. Al-Garadi et al. demonstrated a potent anti-*Salmonella* effect using a plant extract of iscagale lettuce [49], and El-Desoukey et al. demonstrated a potent anti-*Salmonella* effect using an extract of crow liver [50]. Kollanoor-Johny et al. showed that 0.01% cinnamaldehyde and 0.04% eugenol were effective in reducing *Salmonella* invasion in the caecum [51]. Kollanoor Johny et al. described the ability of trans-cinnamaldehyde to effectively reduce *Salmonella* in the caecum [52]. Gurram et al. showed a significant reduction in *Salmonella* in the ileum when fed a mixture of probiotics, chicory, and coriander [53]. Zou et al. found a significant reduction in *Salmonella* in the ileum when broiler chickens were fed a mixture of turmeric, hops, and grape seed extract [54]. Leyva-Diaz et al. found that copper acetate, curcumin, and their combination were effective in reducing *Salmonella typhimurium* colonisation [55]. In our studies, the turmeric additive showed the greatest reduction in *Salmonella* shedding by day 23 post-infection.

The effect of the feed additives on weight gain in the presence of *Salmonella* and *Escherichia coli* infections was significantly different in the turmeric- and enrofloxacin-treated groups than in the control groups only on day 7 after an infection, when sex effects were taken into account. Liu et al. showed significantly better weight gain in broiler chickens fed chicory [56], and Khoobani et al. showed that both groups of broiler chickens fed probiotics and different concentrations of chicory extract had significantly better weight gain compared to the control group. Varmuzova et al. found the same results with the feeding of a feed supplement containing turmeric [42]. Yang et al. showed that fenugreek extract fed to broiler chickens from the third day of life improved daily weight gain [57]. Gurram et al. fed feed supplements containing probiotics, chicory, and coriander, or combinations of these, and found significantly better weight gain in all treatment groups compared to the control group [53]. Zou et al. fed a mixture of turmeric, hops, and grape seed extract to broiler chickens at certain life stages and found significantly better weight gain compared to controls [54]. Our previous studies with So-Ran turmeric also showed significantly better weight gain compared to controls [58]. Kósa et al. also showed the immunomodulatory effect of fermented wheat germ, proven to reduce economic losses [59], but a positive effect on the immune status of pigs has also been described [60].

Significant differences in individual feed consumption were observed between the fenugreek-treated group and the negative control group on day 22 (*p* = 0.0289) and between the turmeric-treated group and the negative control group on day 27 (*p* = 0.0095) and day 29 (*p* = 0.0347). Significant differences in weight gain were observed only at 1 week of age as measured immediately post-infection between the turmeric-treated and positive control groups (*p* = 0.0276) and between the enrofloxacin-treated and positive control groups (*p* = 0.0030). Liu et al. observed similar results in broiler chickens fed chicory [56], and Khoobani et al. and Khoohani et al. showed a significant improvement in the species composition of broiler chickens fed probiotics and different concentrations of chicory extract compared to the control group [61]. However, Gurram et al. found no significant difference in feed intake but a significant improvement in species composition when chickens were fed feed supplements containing probiotics, chicory, and coriander [53].

At post-rearing necropsy, reactive lymph node inflammation was observed in the caecum of all groups, but it was less frequent in the probiotic-, turmeric-, and fenugreek-treated groups compared to the control groups, which could be caused by both *Salmonella* and *Escherichia coli* infection.

In terms of pathological parameters, both villus length and crypt depth were significantly better in the fenugreek-treated group compared to both the positive and negative controls, which, although not significant in terms of absorption surface area alone, did not show significant differences between them. However, when the body weight of the animals tested was taken into account, the fenugreek-treated group and the turmeric-treated group had significantly higher absorption surface areas compared to the positive control group. Yang et al. fed fenugreek seed extract to chickens from the third day of life onwards and found that it increased villus length and the ratio of villus length to crypt depth [57]. Gurram et al. found significantly higher villus length and crypt depth and their ratio in the ileum for all combinations of feed supplements containing probiotics, chicory, and coriander [53]. Another study similarly reported an increase in ileal villus length and crypt depth when probiotics and inulin were combined [62], and in our previous studies, we observed an increase in crypt depth when propolis was fed compared to control groups [63].

## 5. Conclusions

In conclusion, our results showed that the most promising feed supplement, in terms of both natural indicators and tissue parameters, was fenugreek extract. Turmeric extract may also be a worthwhile candidate, and their use in combination should be considered. Further in vivo studies are needed to explore the possible synergistic effect between these two types of extracts when used together. We also plan to conduct a dose-response study to determine the optimal dosage.

## Data Availability

The data presented in this study are in the manuscript and Supplementary Materials.

## Supplementary Materials

The following supporting information can be downloaded at: https://www.mdpi.com/article/10.3390/vetsci10120706/s1.
