# Supplementary material for: Determining the In Vivo Efficacy of Plant-Based and Probiotic-Based Antibiotic Alternatives against Mixed Infection with Salmonella enterica and Escherichia coli in Domestic Chickens
Source: Vet Sci. 2023 Dec 15;10(12):706. doi: 10.3390/vetsci10120706 (PMC10747687; doi:10.3390/vetsci10120706)
Supplement: Supplementary file 1 [file vetsci-10-00706-s001.zip › vetsci-2663211-supplementary.pdf]

Supplementary Table S1: Detailed composition of the pre-starter and starter feed.

Ingredient	Pre-Starter	Starter
Wheat	40.0 %	40.0 %
Soy	29.2 %	31.2 %
Corn	24.8 %	23.8 %
Sunflower seed oil	2.0 %	1.0 %
Premix[$]	4.0 %	4.0 %
Test material[#]	0.1 %	0.1 %

[$] Szőlőfürt Szövetkezet 4% Premix

[#]Added "on-top" of the feed in case of group A, B and C.

Supplementary Table S2: Nutritional parameters of the pre-starter and starter feed.

Name	Prestarter	Starter	Unit
Dry material	88.29	88.17	%
AME poultry-kcal	2 958.00	2 950.63	Kcal
AME poultry-MJ	12.38	12.35	MJ
Raw protein	20.49	18.51	%
Raw fat	4.04	3.17	%
Raw fibre	3.17	2.98	%
ash	5.83	5.59	%
Lysine	1.18	1.05	%
Methionine	0.55	0.52	%
Calcium	0.85	0.83	%
Phosphor	0.66	0.65	%
P-available (avP)	0.43	0.42	%
Sodium	0.17	0.17	%
Vitamin A, added	10 000.00	10 000.00	IE
Vitamin D3, added	5 000.00	5 000.00	IE
Vitamin E, added	85.00	85.00	mg
Zink, added	100.00	100.00	mg
Iodine, added	1.30	1.30	mg
Manganese, added	120.00	120.00	mg
Cupper, added	15.50	15.50	mg
Selenium, added	0.30	0.30	mg
Iron, added	45.00	45.00	mg
SID LYS	1.07	0.95	%
SID MET	0.52	0.49	%
SID SAA	0.81	0.76	%
SID THR	0.77	0.70	%
C18:1	0.81	0.64	%
C18:2	2.11	1.57	%
C18:3	0.08	0.07	%
LA/ALA	27.49	23.25	
6-Phytase	520.00	520.00	FTU

Supplementary Table S3: Summary of the test materials.

Name of test material	Composition	Quantity	Concentration	Manufacturer
Test material A	*Lactobacillus acidophilus*<br>*Lactobacillus plantarum*<br>*Bifidobacterium bifidum*<br>Wheat bran<br>Corncob	$10^{\wedge 7}$ CFU/g<br>$10^{\wedge 7}$ CFU/g<br>$10^{\wedge 11}$ CFU/g<br>40 %<br>Supplemented to 100 %	1 kg/T basal diet	Non commercialized prototype product
Test material B	*Curcuma longa L.*: extract<br>*Triticum aestivum* germ<br>*Cichorium intybus L.* root	1 %<br>10 %<br>Supplemented to 100 %	1 kg/T basal diet	Non commercialized prototype product
Test material C	*Trigonella Foenum graecum* extract<br>Copper (II) chelate of amino acid hydrate<br>*Cichorium intybus L.* root	1 %<br>1.35 %<br>Supplemented to 100 %	1 kg/T basal diet	Non commercialized prototype product

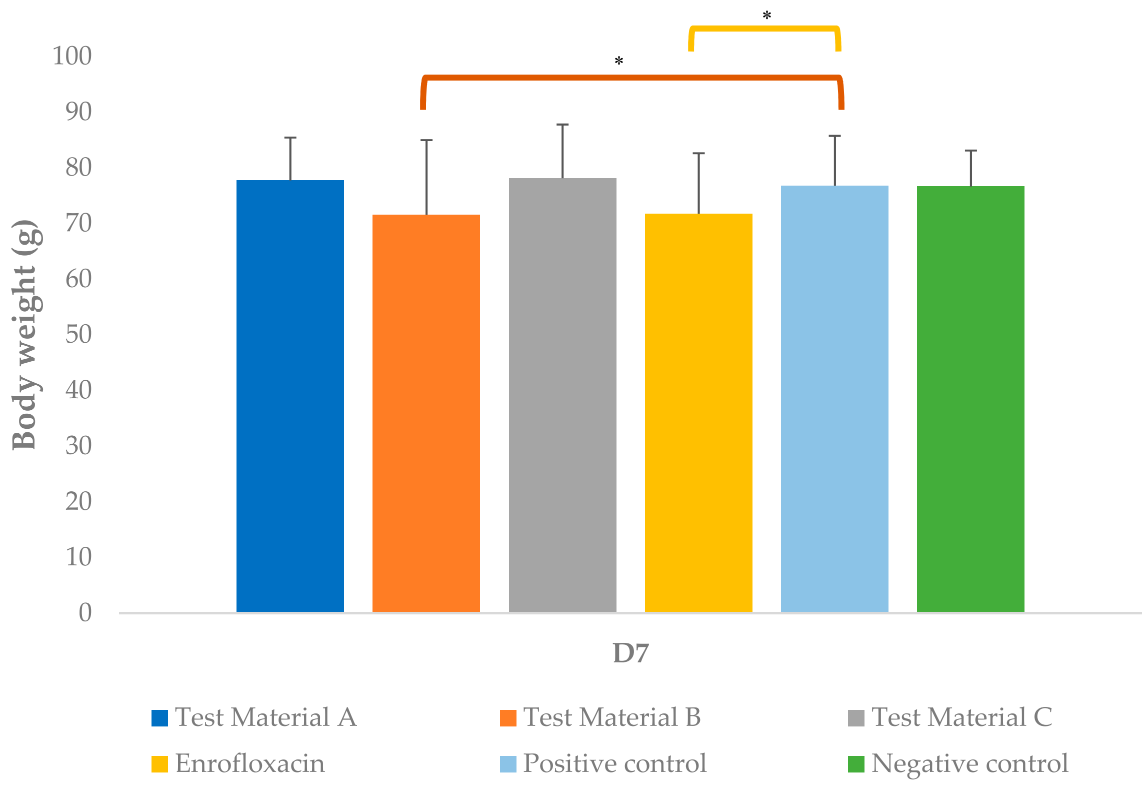

p= 0 ***; 0.001 **; 0.01 *

Supplementary Figure S1: Significant differences in the effect on measurable weight gain after infection. For body weight, we found significant differences between the positive control group and the group treated with test material B (p=0.0224) and between the positive control group and the group treated with enrofloxacin (p=0.0276) only immediately after infection.

Supplementary Table S4: Effect of treatments on body weight on day 7 by statistical analysis compared to positive control group.

**Positive control**	**Estimate**	**Standard Error**	**t value**	**Pr (>|t|)**	
**(Intercept)**	80.6877	1.7834	45.244	< 2e-16	***
Treatment [Test material A]	0.8914	2.3318	0.382	0.7028	
Treatment [Test material B]	-5.6033	2.4299	-2.306	0.0224	*
Treatment [Test material C]	1.1025	2.4024	0.459	0.6469	
Treatment [Enrofloxacin]	-5.3847	2.3328	-2.308	0.0276	*
Treatment [Negative control]	-0.1976	2.3116	-0.085	0.9320	
Sex [Female]	-7.7295	1.3804	-5.599	<0.0001	***

Significance codes: 0 '***' 0.001 '**' 0.01 '*' 0.05 '.' 0.1 ' ' 1

Supplementary Table S5: Differences in villus length per treatment compared to positive and negative control groups by statistical analysis.

**Positive control**	**Estimate**	**Standard Error**	**t value**	**Pr (>|t|)**	
**(Intercept)**	639.582	34.584	18.494	< 2e-16	***
Treatment [Test material A]	20.178	37.865	0.533	0.59535	
Treatment [Test material B]	14.157	41.718	0.339	0.73510	
Treatment [Test material C]	123.058	41.312	2.979	0.00367	**
Treatment [Enrofloxacin]	51.677	40.183	1.286	0.20155	
Treatment [Negative control]	94.701	40.078	2.363	0.02017	*
Sex [Female]	-5.798	26.268	-0.221	0.82578	
**Negative control**	**Estimate**	**Standard Error**	**t value**	**Pr (>|t|)**	
**(Intercept)**	734.283	28.170	26.066	<2e-16	***
Treatment [Test material A]	-74.523	40.078	-1.859	0.0661	.
Treatment [Test material B]	28.357	39.751	0.713	0.4774	
Treatment [Test material C]	-80.544	38.117	-2.113	0.0372	*
Treatment [Enrofloxacin]	-43.024	40.326	-1.067	0.2887	
Treatment [Positive control]	-94.701	40.078	-2.363	0.0202	*
Sex [Female]	-5.798	26.268	-0.221	0.8258	

Significance codes: 0 '***' 0.001 '**' 0.01 '*' 0.05 '.' 0.1 ' ' 1

Supplementary Table S6: Differences in crypt depth per treatment compared to positive and negative control groups by statistical analysis.

**Positive control**	**Estimate**	**Standard Error**	**t value**	**Pr(>|t|)**	
**(Intercept)**	120.5484	6.6839	18.036	<2e-16	***
Treatment [Test material A]	-6.6500	7.3179	-0.909	0.36579	
Treatment [Test material B]	4.3958	8.0627	0.545	0.58690	
Treatment [Test material C]	23.1975	7.9842	2.905	0.00456	**
Treatment [Enrofloxacin]	1.2505	7.7660	0.161	0.87242	
Treatment [Negative control]	0.4843	7.7457	0.063	0.95027	
Sex [Female]	6.3353	5.0768	1.248	0.21514	
**Negative control**	**Estimate**	**Standard Error**	**t value**	**Pr(>|t|)**	
**(Intercept)**	121.0327	5.4443	22.231	<2e-16	***
Treatment [Test material A]	-7.1343	7.7457	-0.921	0.35935	
Treatment [Test material B]	3.9114	7.3667	0.531	0.59668	
Treatment [Test material C]	22.7132	7.6826	2.956	0.00393	**
Treatment [Enrofloxacin]	0.7661	7.7936	0.098	0.92190	
Treatment [Positive control]	-0.4843	7.7457	-0.063	0.95027	
Sex [Female]	6.3353	5.0768	1.248	0.21514	

Significance codes: 0 '***' 0.001 '**' 0.01 '*' 0.05 '.' 0.1 ' ' 1

Supplementary Table S7: Differences in villus width per treatment compared to positive and negative control groups by statistical analysis.

**Positive control**	**Estimate**	**Standard Error**	**t value**	**Pr(>|t|)**	
**(Intercept)**	109.368	6.409	17.066	<2e-16	***
Treatment [Test material A]	-15.700	7.016	-2.238	0.0276	*
Treatment [Test material B]	-7.087	7.731	-0.917	0.3616	
Treatment [Test material C]	-10.619	7.655	-1.387	0.1686	
Treatment [Enrofloxacin]	-0.715	7.446	-0.096	0.9237	
Treatment [Negative control]	-12.333	7.426	-1.661	0.1001	
Sex [Female]	1.045	4.868	0.215	0.8304	

Significance codes: 0 '***' 0.001 '**' 0.01 '*' 0.05 '.' 0.1 ' ' 1
